# Medical Students

**Published:** 1849-05

**Authors:** 


MEDICAL STUDENTS.

The number of students in attendance at the Medical College of Ohio during the recent session, was 175, the number of graduates 50; at the Rush Medical College, in Chicago, the number of students was 100, and the number of graduates 18. The class at the College of Physicians and Surgeons of New York numbered 178; that at the Georgia Medical College 133, of whom 36 received the degree of M.D. The largest graduating class in this country was the late one at the University of Pennsylvania, and amounted to 190. The number graduated at the Jefferson Medical College was 188. The classes in both these schools were large.

